# The Overexpression of miR-377 Aggravates Sepsis-Induced Myocardial Hypertrophy by Binding to Rcan2 and Mediating CaN Activity

**DOI:** 10.1155/2022/6659183

**Published:** 2022-10-11

**Authors:** Shiji Wang, Guang Wang, Lihua Dong, Xingang Liu, Weiyun Pan, Jinfeng Han, Ying Lu

**Affiliations:** Department of Critical Care Medicine, The First Bethune Hospital of Jilin University, Changchun 130021, China

## Abstract

Sepsis remains a complicated and incompletely understood syndrome, and myocardial dysfunction is one of the main complications contributing to poor clinical outcomes. Accumulating evidence has revealed the critical involvement of the deregulated expression of specific microRNAs (miRNAs) in cardiac pathologies caused by sepsis. Intriguingly, miR-377 has been correlated with cardiomyocyte apoptosis, whereas its effect on myocardial hypertrophy remains to be illustrated. Thus, the current study sets out to explore the impact and underlying mechanism of miR-377 on myocardial hypertrophy induced by sepsis. The expression pattern of miR-377 was detected in myocardial tissues of septic mice induced by cecal ligation-perforation (CLP). We found that miR-377 was highly expressed in myocardial tissues of CLP-induced septic mice with cardiomyocyte hypertrophy. Besides, miR-377 inhibition could relieve cardiomyocyte hypertrophy and reduce inflammation in septic mice. Further, mechanistic studies found that miR-377 could target Rcan2 and then regulate calcineurin (CaN) activity via Ca^2+^/CaN signaling pathway. Collectively, our findings illuminate that miR-377 enhances myocardial hypertrophy caused by sepsis, by targeting Rcan2 and further regulating the Ca^2+^/CaN signaling pathway. This work highlights downregulation of miR-377 as a novel target for the management of sepsis-induced myocardial hypertrophy.

## 1. Introduction

Sepsis, a common disease accompanied by high morbidity and mortality rates, is an inflammatory response syndrome resulting from the invasion of pathogenic microorganisms [1]. The toxins and metabolites produced by bacterial, fungal, or viral infection invade into the blood and consequently activate cells and immune system to rapidly augment the production of cytokines and endogenous mediators [2]. Clinically, sepsis is often complicated with acute organ dysfunction, which remains one of the leading causes of death in sepsis patients [3]. It is notable that myocardial tissues are the main target organ in the progression of sepsis, and the damage of myocardial tissue is generally regarded as the commencement of various organ dysfunction syndromes [4]. Moreover, numerous studies have revealed that sepsis has a negative effect to confer on cardiomyocytes, which is an important contributor to hemodynamic instability and cardiac insufficiency [5–7]. In this sense, it would be prudent to investigate the unclear mechanism of sepsis on myocardial injury to further explore the potential targets and biomarkers for sepsis treatment.

microRNAs (miRNAs), small RNAs with 20-24 nucleotides, possess the ability to restrict gene expression by affecting mRNA degradation or blocking translation [8]. Furthermore, miRNAs play critical roles in the development and maintenance of heart physiological functions, with various miRNAs being highlighted as mediators or therapeutic targets for heart failure [9]. In addition, dysregulation of miRs has been implicated in various biological processes related to cardiovascular diseases [10, 11]. One such miRNA, miR-377, targeting many genes, was previously indicated to be highly expressed in disorders associated with immune response [12, 13]. Further, miR-377 has been suggested to mediate cardiomyocyte apoptosis induced by cyclosporin A [14]; yet, its role in cardiomyocyte hypertrophy caused by sepsis remains to be investigated.

Initial bioinformatic prediction indicated regulators of calcineurin 2 (Rcan2) as a target gene of miR-377. RCANs, also known as calcipressin, are regarded as pivotal regulators of different cellular processes, such as muscle fiber remodeling and immune response [15]. In addition, RCAN proteins were previously associated with the progression of several pathological conditions, including cardiac hypertrophy [16]. Mechanistically, RCANs play roles in physical binding and regulation of the Ca^2+^ and calmodulin-dependent serine-threonine phosphatase calcineurin (CaN) [16, 17]. Furthermore, Ca^2+^/CaN signaling pathway is crucial for regulating the function of cardiomyocytes. For instance, aspirin impeded cardiac hypertrophy through inhibition of the Ca^2+^/CaN signaling pathway in vitro and in vivo [18]. On the basis of the aforementioned lines of evidence, the current study is aimed at investigating the regulatory effect of miR-377 on the cardiomyocytes of CLP-induced septic mice and further explored its downstream active pathways.

## 2. Materials and Methods

### 2.1. Ethics Statement

Animal experimentation protocols were approved by the Animal Care and Use Committee of the First Bethune Hospital of Jilin University, and all procedures were in compliance with the Guide for the Care and Use of Laboratory Animals.

### 2.2. Mice Model Preparation, Grouping, and Treatment

Kunming (KM) male mice (weighing 25-30 g, aged 6-8 weeks) were provided by the Cyagen Biosciences Inc. (Guangzhou, China), and all mice were raised under specific pathogen-free (SPF) conditions. After 3-5 days of acclimatization, the mice were subjected to cecal ligation-perforation (CLP) to establish a mouse model of sepsis. Briefly, all the mice were fasted for 12 h presurgery, with free access to water. Then, the mice were intraperitoneally anesthetized with 3% pentobarbital sodium (3 mL/kg) and fixed in the supine position. After routine skin preparation and disinfection, the cecum was separated by laparotomy at 1.5 cm along the midline of the abdomen. Subsequently, a 2-0 silk thread was used to ligate the end of cecum, and a No. 18-gauge needle was employed for ligation for 3 times. The intestinal feces were squeezed out, and then the incision was closed with layered suture.

Afterwards, the mice were divided into 8 groups, 9 mice per group. Untreated mice were set as the control group, and sham-operated mice (only treated with cecum exposure and abdominal suture incision) were referred to as the sham group. Mice of the agomir negative control (NC), antagomir NC, micro-RNA- (miR-) 377 agomir, and miR-377 antagomir groups were injected with the plasmids (5 mg/mL) expressing corresponding molecules via caudal vein and further subjected to CLP 4 days later; mice of other two groups were injected in the same way with the combination of miR-377 agomir plasmids and Rcan2 overexpression plasmids (oe-Rcan2) or the combination of miR-377 antagomir plasmids and plasmids carrying small interfering RNA (siRNA) targeting Rcan2 (si-Rcan2), referred to as the miR-377 agomir + oe-Rcan2 group and the miR-377 antagomir + si-Rcan2 group. The miRNA agomir (miR40003123-4-5) and miRNA antagomir (miR30003123-4-5) used in the present study were purchased from the RiboBio company (Guangzhou, Guangdong, China).

The lentiviral vector was constructed by Shanghai GenePharma Co., Ltd. (Shanghai, China) and packaged in HEK293T cells obtained from American Type Culture Collection (ATCC Rockville, Maryland). HEK293T cells were cultured in RPMI-1640 with 10% fetal calf serum (FBS) and passaged every other day. The lentiviral vectors were collected and diluted to a density of 1 × 10^9^ pfu/100 *μ*L, and then 10 *μ*L of lentiviral vector was slowly injected into the vein of the anesthetized mice with a 27-gauge needle and processed for three consecutive days. Following that, 7 mice from each group were adopted for the experiment. After the successful development of mouse models of different groups, blood samples were drawn from the inferior vena cava and allowed to stand for 2 h. Afterward, the blood sample was centrifuged at 2000 r/min for 20 min, and the supernatant was obtained and stored at -80°C. In addition, the ventricular muscle tissues were harvested, quick-frozen in liquid nitrogen, and stored at -80°C for subsequent index detection.

### 2.3. Heart Index Measurement

Echocardiography was performed at 8 weeks after the operation to examine the thickness of left ventricle posterior wall (LVPW), the left ventricular end-systolic diameter (LVESD), ejection fraction (EF), and fractional shortening (FS). The heart of mouse was taken out, rinsed with phosphate-buffered saline (PBS) buffer, and wiped with filter paper. Subsequently, the heart weight (HW) and left ventricle weight (LVW) were measured by an electronic balance, and the ratio of LVW to body weight (LVW/BW) was calculated.

### 2.4. ELISA

According to the instructions of the ELISA kit (R&D System, Minneapolis, MN), the levels of serum cardiac troponin I (CTn-I) and brain natriuretic peptide (BNP) were measured. Moreover, the concentration of interferon gamma (IFN-*γ*), tumor necrosis factor alpha (TNF-*α*), interleukin IL-6 and interleukin IL-8, and other cytokines were also quantified using corresponding ELISA kits.

### 2.5. Hematoxylin and Eosin (HE) Staining

HE staining was employed to observe the structure of cardiomyocytes. After fixing the myocardial tissue with 4% paraformaldehyde for 24 hours, the sections were deparaffinized and stained with HE for 10 min. Then, the sections were successively treated with 1% hydrochloric acid ethanol and 2% sodium bicarbonate and immersed in eosin staining solution. Following dehydration with gradient alcohol, the sections were sealed with neutral resin and photographed after drying for 72 h. Subsequently, about 1 mm^3^ of myocardial tissue was fixed with 2.5% glutaraldehyde solution and 1% osmium acid, then dehydrated with ethanol and embedded with epoxy resin Epon 812. Later, an ultramicrotome (Olympus, Tokyo, Japan) was adopted to slice the abovementioned tissue. The slices were stained by uranyl acetate and lead citrate, and then the ultrastructural changes of cardiomyocytes were observed under a transmission electron microscope (Hitachi, Tokyo, Japan).

### 2.6. Isolation and Culture of Primary Cardiomyocyte

Mice aged 1 to 3 days were fixed on the foam board under aseptic conditions, and the surfaces of mice and foam board were disinfected with alcohol. The chest cavity along the 3rd and 4th intercostal space of mice was opened, and the ventricles were extracted and placed in a petri dish with precooled PBS. Afterwards, the large blood vessels and other nonmyocardial tissues were removed, and the ventricles were sliced into 1 mm^3^ blocks on ice. After adding 5 times of the volume of pancreatin and collagenase mixture (1 : 1), the tissue blocks were digested at 37°C for 10 min, and the mixture was shaken every 2 min. Later, the tissue blocks were pipetted, and the digestion step was repeated. The cell suspension was collected in a 15 mL centrifuge tube and then added equal amounts of DMEM/F12 medium containing 20% FBS to terminate the digestion. Following 10 min centrifugation at 800 rpm, the supernatant was discarded, and a single cell suspension was obtained by pipetting. After passing a 200-mesh sieve, the cardiomyocytes were purified by differential adherence to remove noncardiomyocytes. The nonadherent cell suspension was transferred to another culture flask, and 5′-bromodeoxyuridine (0.1 mmol/L) was added to inhibit the growth of noncardiomyocytes. Cells were seeded into plates at a density of 5 × 10^5^, cultured at 37°C, 5% CO_2_, and saturated humidity.

### 2.7. Cell Grouping and Transfection

After 48 h of culture, the primary cardiomyocytes were exposed to Ang II at a final concentration of 10^6^ mol/L for 24 h and then classified into the sham group (received no further treatment) and experimental groups, wherein the Ang II-treated cells were, respectively, transfected with plasmids expressing mimic NC, miR-377 mimic, inhibitor NC, miR-377 inhibitor, miR-377 inhibitor + si-Rcan2, and oe-miR-377 + oe-Rcan2. All the aforementioned plasmids were purchased from Dharmacon, Inc. (Lafayette, CO).

Briefly, cells were seeded in a 6-well plate at a density of 3 × 10^5^/well, and upon reaching 80% confluence, the cell transfection was conducted with Lipofectamin 2000 kits (Invitrogen) (Thermo Fisher Scientific, Waltham, MA). For the transfection, 4 *μ*g of the target plasmids and 10 *μ*L of Lipofectamine 2000 were separately diluted with 250 *μ*L of serum-free Opti-MEM medium (Gibco, Grand Island, NY), mixed and placed in a 37°C, 5% CO_2_ incubator. Then, cells were cultured with complete medium after 6 h and collected after 48 h of continuous culture.

### 2.8. Quantitative Real-Time Polymerase Chain Reaction (qRT-PCR)

Total RNA content was extracted from myocardial tissues and cells using the TRIzol Reagent (Takara Bio, Shiga, Japan) for detection of miR and mRNA levels. The primer sequences of miR-377, Rcan2, CaN, U6, and *β*-actin were designed and synthesized by Invitrogen (Carlsbad, CA), as presented in Supplementary Table 1. U6 and *β*-actin were separately used as the internal reference for miR-377 and Rcan2. For miRNA quantification, cDNA was obtained with poly(A) tailing method using a miRNA reverse transcription kit (D1801, HaiGene, Harbin, China); for mRNA quantification, 2 *μ*g RNA was reversely transcribed into cDNA using the ReverTra Ace qPCR RT Kit (TOYOBO, Osaka, Japan). Then, RT-qPCR was performed with the help of SYBR Green PCR Master Mix kit (Roche, Indianapolis, IN), and the expression of target genes was calculated according to the 2^-*ΔΔ*Ct^ method.

### 2.9. Western Blot Analysis

Total proteins were extracted from tissues and cells by RIPA lysate (Beyotime Biotechnology, Shanghai, China) containing 1% phenylmethylsulfonyl fluoride (PMSF), followed by the determination of protein concentration using the bicinchoninic acid (BCA) kit (Beyotime Biotechnology, Shanghai, China). The extracted protein was separated by 10% polyacrylamide gel electrophoresis and then transferred onto PVDF membrane (Merck Millipore, Massachusetts). Subsequently, the membrane was blocked with 5% bovine serum albumin (BSA) for 1 h and then incubated with rabbit anti-mouse primary antibody (Rcan2, 1 : 1000, 254029, Abbiotec, San Diego, CA; CaN, 1 : 500, PAB8606, Abnova, Walnut, CA) overnight at 4°C. After washing three times with TBST buffer, goat anti-rabbit secondary antibody conjugated to peroxidase (1 : 1000, A0208, Beyotime Biotechnology, Shanghai, China) was added to incubate above membrane at room temperature for 1 h. Furthermore, the protein bands were visualized with enhanced chemiluminescence (ECL) reagent, and the gray value of the target protein band was analyzed using the ImageJ software, with *β*-actin serving as an internal reference.

### 2.10. Bioinformatic Analysis and Determination of Dual Luciferase Activity

The microarray GSE9667 related to sepsis-induced cardiomyocyte hypertrophy was retrieved from the Gene Expression Omnibus (GEO) database, and the affy package in *R* language was adopted to standardize the expression data. Additionally, the target genes of miR-377, the target relationship between miR-377 and Rcan2, and the binding sites of miR-377 and Rcan2 3′UTR were analyzed using a biological prediction website (http://www.microrna.org/).

Next, the promoter region of Rcan2 was constructed into pGL3-Basic vector (Promega, Madison, WI), as the pGL3-Rcan2 recombinant vector. Then, HEK293T cells were seeded in 24-well plates at a density of 3 × 10^4^/well. Based on site-directed mutagenesis method, the Rcan2 3′-UTR fragment with site mutation was constructed and inserted into the pGL3-Basic vector, and the inserted sequence was verified by sequencing. Using the liposome transfection method, pGL3-Rcan2 or pGL3-mut Rcan2 was, respectively, cotransfected with mimic NC, miR-377 mimic, inhibitor NC, and miR-377 inhibitor into HEK293T cells, and the Renilla plasmid was simultaneously transfected as reference. After 48 h of transfection, the luciferase reporter gene detection was carried out using a dual luciferase reporter gene analysis system (Promega, Madison, WI). Luminescence intensity was examined by the multifunctional microplate reader SpectraMaxM5 (Molecular Devices, Shanghai, China), with Renilla luciferase as the internal reference gene.

### 2.11. Evaluation of [Ca^2+^]_*i*_ Concentration

The cardiomyocytes loaded with fura-2 were placed under a fluorescence microscope, with the excitation wavelength of 340/380 nm and an emission wavelength of 510 nm. Following, the fluorescence signal was processed using the Felix software. The concentration of [Ca^2+^]*i* was calculated with the formula provided by Gynkiewicz: [Ca^2+^]_*i*_ = [(*R*−−Rmin)/(Rmax−−*R*)] × (Sf/Sb) × Kd. Among them, Kd was 224 nmol/L, *R* represented the fluorescence value, Sf indicated free calcium fluorescence intensity, Sb was bound calcium fluorescence intensity, and [Ca^2+^]_*i*_ concentration unit was nmol/L.

### 2.12. The Activity of CaN

To detect the CaN activity, cardiomyocytes were washed twice with ice-cold Tris-HCl buffer and added with 1 mL of homogenization buffer for protein phosphorylation. Cells were collected and placed on the ice for 10 min, and meanwhile, 22 g needles were adopted to promote lysis. Following centrifugation at 20000 g for 10 min at 4°C, the supernatant was harvested, and a small amount of supernatant was used to measure the protein concentration. The activity of CaN was measured according to the instructions of the CaN assay kit.

### 2.13. Cell Proliferation Assay

Cell proliferation was examined utilizing the 5-ethynyl-2′-deoxyuridine (EdU) method. Cardiomyocytes at the logarithmic phase of growth were plated at a density of 4 × 10^3^ to 1 × 10^5^ and cultured to normal growth stage. According to the kit instructions, the EdU proliferation detection kit was used to measure cell proliferation of each group.

### 2.14. Flow Cytometry Analysis of Apoptosis

After 48 h of transfection, the cells were digested by trypsin without EDTA and collected. Subsequently, the AnnexinV-FITC Apoptosis Detection Kit (CA1020, Beijing Solarbio Science & Technology Co., Ltd., Beijing, China) was employed to detect cell apoptosis. After washing with binding buffer, the cells were resuspended in the mixture of Annexin-V-FITC and binding buffer (1 : 40), shaken for mixture, and then incubated at room temperature for 30 min. Then, the mixture of propidium iodide (PI) and binding buffer was added to above solution and incubated for 15 min after mixing. Later, the cell apoptosis was measured using a flow cytometer.

### 2.15. Statistical Analysis

The statistical analyses were performed using the SPSS 21.0 (IBM Corp. Armonk, NY). Measurement data were expressed as mean ± standard deviation. Unpaired *t*-test was adopted to analyze the unpaired data in two groups. Data comparisons between multiple groups were performed by one-way analysis of variance (ANOVA) with Tukey's posthoc test. A value of *p* < 0.05 was considered statistically significant.

## 3. Results

### 3.1. miR-377 Is Highly Expressed in Myocardial Tissues of Mouse Models wherein Sepsis Was Successfully Induced by CLP

It has been reported that miR-377 was overexpressed in disorder associated with immune response [13], but its expression in septic cardiomyocytes is yet to be reported.

**Figure 1:**
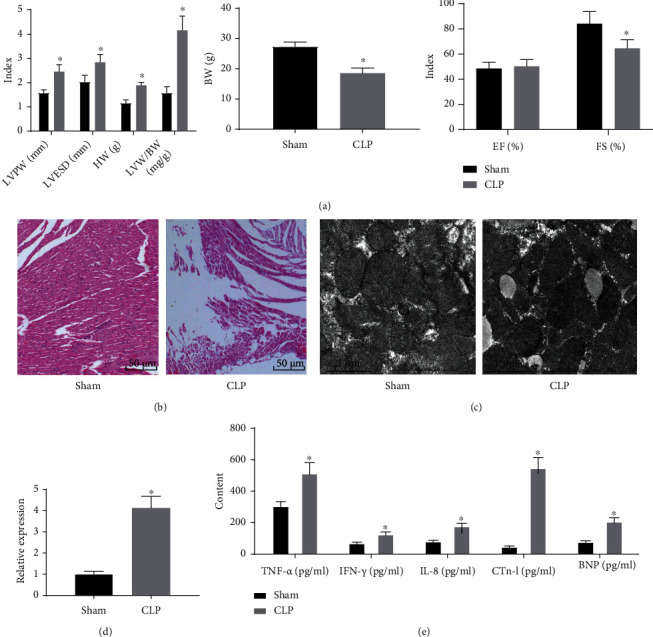
MiR-377 is highly expressed in myocardial tissues of mice with CLP-induced sepsis. (a) Evaluation of cardiomyocyte hypertrophy in CLP-induced septic mice by echocardiography and ventricular body mass ratio (*n* = 9), ^∗^*p* < 0.05 compared with sham-operated mice. (b) HE staining of myocardial tissues of mice in sham-operated mice and CLP-induced septic mice group. (c) Myocardial tissue morphology of mice in sham-operated mice and CLP-induced septic mice, as observed by transmission electron microscope. (d) The expression level of miR-377 in the myocardial tissues in sham-operated mice and CLP-induced septic mice, as measured by qRT-PCR. ^∗^*p* < 0.05 compared with sham-operated mice. (e) The level of inflammatory factors and serum myocardial enzyme indexes of myocardial tissues in sham-operated mice and CLP-induced septic mice, as examined by ELISA. ^∗^*p* < 0.05 compared with sham-operated mice. Measurement data were showed as *mean* ± *standard* *deviation*. Data of two-group were analyzed by unpaired *t*-test.

Herein, we established a mouse model of sepsis to explore the expression of miR-377 in septic cardiomyocytes. As compared with sham-operated mice, CLP-treated presented with signs of lethargy, poor response, less activity, erect hair, chills, little will to drinking, low appetite, increased secretions at the corners of the eyes, and morbidity became more obvious over time. After 8 weeks of model establishment, the results of echocardiography displayed upregulated indices of LVPW, LVESD, HW, and LVW/BW as well as down-regulated indices of BW and FS in mouse models, relative to sham-operated mice (Figure 1(a)).

Moreover, the myocardial tissues in sham-operated mice were in normal conditions, without edema, degeneration, and atrophy, as illustrated by HE staining. In contrast, CLP-induced mice presented with more inflammatory cell infiltration, mononuclear neutrophil changes, inflammatory cells, and myocardial degeneration of small blood vessel stasis (Figure 1(b)). Furthermore, results of transmission electron microscopy revealed that the in the sham-operated mice, the nucleus structure was complete; nucleus was uniformly stained; the myocardial T tube and sarcoplasmic reticulum were few but not dilated; the extrafascicular matrix exhibited no edema and penetration; the mitochondria were neatly arranged, with large and clear cristae; and vascular endothelial cells had moderate cell vesicles, whereas, oncolysis, edema, interstitial inflammation, enlargement of myocardial T-tube, and sarcoplasmic reticulum were observed in part of myocardial sarcoplasm in the CLP-induced mice, and myocardial mitochondria proliferated under the sarcoplasmic line were observed in the CLP-induced mice (Figure 1(c)).

In addition, qRT-PCR results illustrated that in relative to that in sham-operated mice, miR-377 was overexpressed in the myocardial tissues of CLP-treated mice (Figure 1(d)). Results of ELISA assay showed that levels of TNF-*α*, IL-6, and IL-8 in myocardial tissues, as well as CTn-I and BNP in serum, were higher in mice of the CLP-induced mice versus the sham-operated mice (Figure 1(e)). These data collectively supported the successful establishment of the mouse model of sepsis and confirmed that miR-377 was highly expressed in myocardial tissues of CLP-induced septic mouse.

### 3.2. MiR-377 Inhibition Relieves CLP-Induced Septic Mice with Cardiomyocyte Hypertrophy

**Figure 2:**
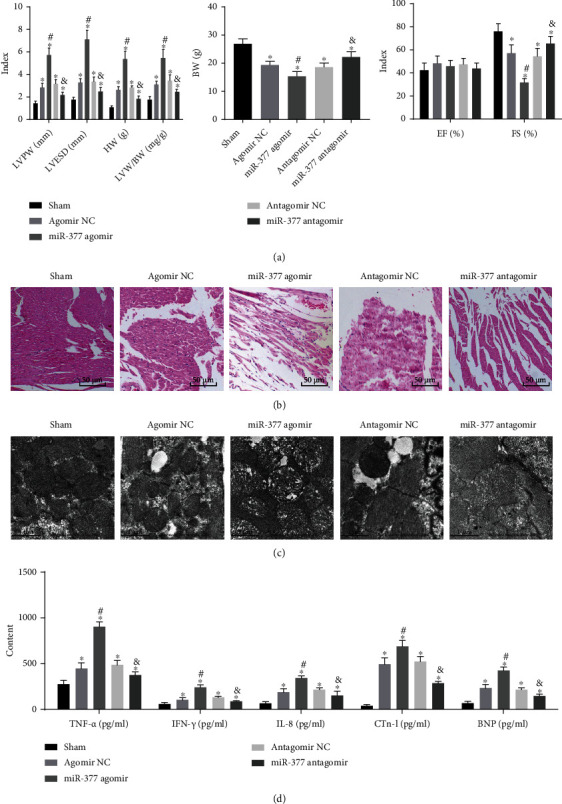
MiR-377 repression could ameliorate cardiomyocyte hypertrophy in septic mice induced by CLP. (a) LVPW, LVESD, EF, and FS detected by echocardiography and HW and LVW/BW measured by weighing, reflecting the successful establishment of a sepsis mouse model, wherein the sepsis was induced by CLP. (b) Myocardial damage of mice in each group, as detected by HE staining. (c) Myocardial tissue morphology of mice in each group, as observed by transmission electron microscope. (d) The expression of inflammatory factors in myocardial tissues of mice, as evaluated by ELISA assay. ^∗^*p* < 0.05 compared with sham-operated mice. #*p* < 0.05 compared with the antagomir NC group. &*p* < 0.05 compared with the antagomir NC group. Measurement data were represented as *mean* ± *standard* *deviation*. One-way ANOVA with Tukey's posthoc test was employed to analyze data of multiple groups. *n* = 9, mice in each group.

Further, to elucidate the role of miR-377 in cardiomyocyte hypertrophy of septic mice, we performed lentiviral transfection to overexpress or inhibit the miR-377 expression. According to results of echocardiography and LVW/BW measurement, CLP induction could lead to elevated LVPW, LVESD, HW, and LVW/BW as well as reduced BW and FS, as compared with sham-operated mice. These effects were further strengthened in response to miR-377 agomir, while the presence of miR-377 antagomir resulted in diminished LVPW, LVESD, HW, and LVW/BW as well as elevated BW and FS (Figure 2(a)). The results of HE staining and electron microscope observation demonstrated the increased damage of myocardial ultrastructure, the expansion of sarcoplasmic reticulum and plasma reticulum, and obvious infiltration of inflammatory cell in CLP-induced septic mice in response to the miR-377 overexpression. In addition, miR-377 inhibition significantly alleviated the symptoms of myocardial injury in mice and its myocardial tissue inflammation (Figures 2(b) and 2(c)).

Subsequently, the focus shifted to validate whether miR-377 was involved in the inflammatory response induced by CLP. The results of ELISA assay showed that in relative to the sham-operated mice, the expression of cytokines (including IL-6, IFN-*γ*, IL-8 and TNF-*α*) was enhanced in the CLP-induced mice. Besides, the levels of these cytokines were observed to be elevated in response to the miR-377 overexpression, while being reduced in response to miR-377 silencing (Figure 2(d)). These data indicated that suppressing miR-377 could alleviate cardiomyocyte hypertrophy in septic mice.

### 3.3. Rcan2 Is Poorly Expressed in Myocardial Tissues of Septic Mice and Is a Specific Target Gene of miR-377

**Figure 3:**
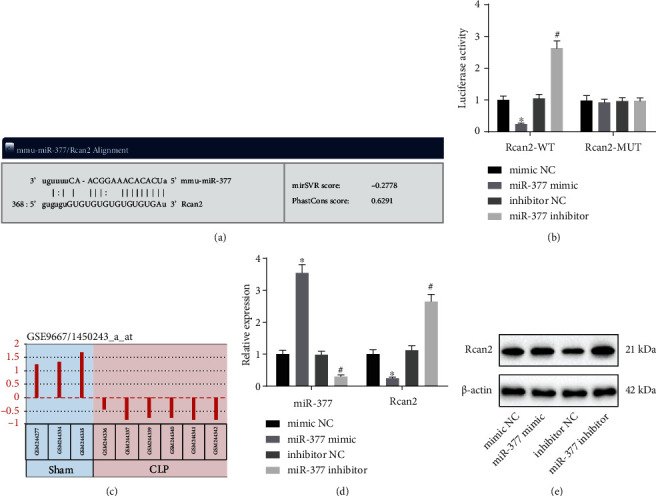
MiR-377 negatively regulated Rcan2 level (*n* = 9). (a) The sequence of 3′-UTR region of miR-377 and Rcan2 gene. (b) The results of double luciferase reporter gene experiment. (c) Heat map analyzed by GSE9667 microarray dataset. (d) The level of miR-377 and Rcan2 in hypertrophic myocardial tissue of mice, as measured by qRT-PCR. (e) The expression of Rcan2 in hypertrophic myocardial tissue of mice, as detected by Western blot. ^∗^*p* < 0.05 compared with the mimic NC group. #*p* < 0.05 compared with the inhibitor NC group. Measurement data were expressed as *mean* ± *standard* *deviation*. The data comparison between multiple groups was performed by ANOVA. Unpaired *t*-test was adopted to analyze the data between two groups. Above experiments were repeated in triplicate.

Furthermore, the sequence of 3′-UTR region of Rcan2 gene targeted by miR-377 was predicted using a biological prediction website (http://www.microRNA.org) (Figure 3(a)). Subsequent luciferase activity assay demonstrated that the luminescence signal decreased after transfection of miR-377 mimic and Rcan2-Wt, while the transfection of miR-377 inhibitor and Rcan2-Wt augmented the luminescence signal. Meanwhile, the luciferase intensity of each group transfected with Rcan2-Mut exhibited no significant difference (Figure 3(b)). These results implied that miR-377 could specifically bind to Rcan2.

Profiling of the GSE9667 microarray dataset further suggested that Rcan2 was poorly expressed in the myocardium of septic mice induced by CLP (Figure 3(c)). Thus, we focused our efforts to investigate the expression of Rcan2 in cardiomyocyte hypertrophy of septic mice. The miR-377 expression was enhanced, while Rcan2 level was downregulated in response to miR-377 mimic, as measured by qRT-PCR and Western blot. Meanwhile, the levels of miR-377 and Rcan2 showed opposing trends in response to miR-377 inhibitor (Figures 3(d) and 3(e)). Taken together, these findings revealed that miR-377 targeted and negatively regulated Rcan2.

### 3.4. MiR-377 Regulates CaN Activity by Inhibiting Rcan2

**Figure 4:**
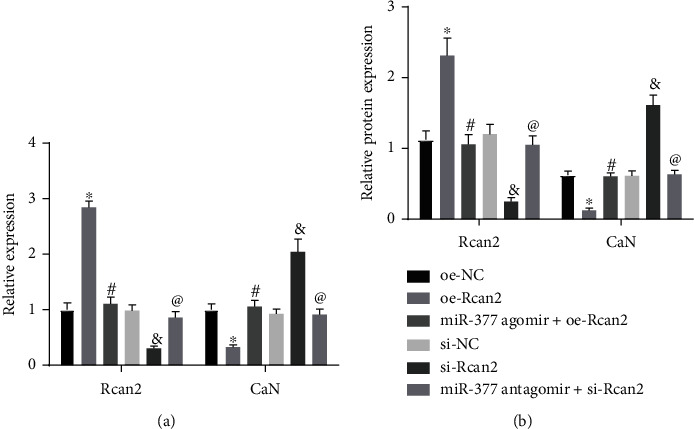
MiR-377 modulated CaN activity by restricting Rcan2 (*n* = 9). (a) The mRNA level of Rcan2 and CaN in hypertrophic myocardial tissues of mice, as examined by qRT-PCR. (b) The protein expression of Rcan2 and CaN in hypertrophic myocardial tissues of mice, as measured by Western blot. ^∗^*p* < 0.05 compared with the oe-NC group. #*p* < 0.05 compared with the oe-Rcan2 group. &*p* < 0.05 compared with the si-NC group. @*p* < 0.05 compared with the si-Rcan2 group. Measurement data were represented as *mean* ± *standard* *deviation*. Comparisons between multiple groups were performed by one-way ANOVA, followed by Tukey's posthoc test. Above experiments were repeated in triplicate. *n* = 9, mice in each group.

Rcan2 is known to ameliorate the hypertrophy of cardiomyocytes by suppressing CaN activity [19]. The results of qRT-PCR and Western blot assays demonstrated that the Rcan2 expression was enhanced, and the CaN expression was reduced in response to oe-Rcan2. Meanwhile, combined treatment with miR-377 agomir and oe-Rcan2 was found to decrease the Rcan2 level and promote the CaN expression. Moreover, si-Rcan2 diminished the Rcan2 level and improved the CaN expression. Additionally, the Rcan2 expression was promoted, and CaN level was reduced in response to miR-377 antagomir and si-Rcan2 (Figure 4). Overall, these findings suggested that miR-377 mediated CaN activity through targeted inhibition of Rcan2.

### 3.5. Ca^2+^-CaN Signaling Pathway Promotes Cardiomyocyte Hypertrophy in Septic Mice Induced by CLP

**Figure 5:**
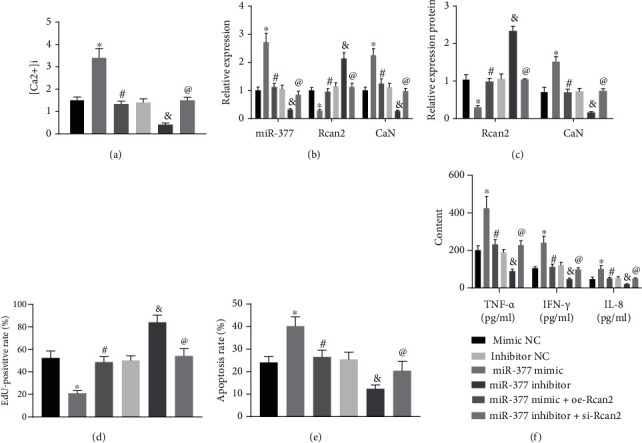
MiR-377 increased CLP-induced cardiomyocyte hypertrophy in septic mice by inhibiting Rcan2 and regulating CaN activity through Ca^2+^-CaN pathway. (a) [Ca^2+^]_*i*_ in cardiomyocytes of each group. (b) The level of miR-377, Rcan2, and CaN, as measured by qRT-PCR. (c) The protein expression of Rcan2 and CaN, as examined by Western blot. (d) The result of cell proliferation in each group as examined by EdU assay. (e) The result of cell apoptosis in each group as examined by flow cytometry. (f) The expression level of inflammatory factors (TNF-*α*, IL-6, and IL-8) quantified by ELISA assay. ^∗^*p* < 0.05 compared with the mimic NC group. #*p* < 0.05 compared with the miR-377 mimic group. &*p* < 0.05 compared with the inhibitor NC group. @*p* < 0.05 compared with the miR-377 inhibitor group. Measurement data were showed as *mean* ± *standard* *deviation*. Data of multiple groups were compared by one-way ANOVA with Tukey's posthoc test. All experiments were repeated for 3 times.

Following the aforementioned findings, we then explored the role of Ca2^+^-CaN signaling pathway in sepsis-induced myocardial injury. We measured the calcium ion concentration ([Ca^2+^]_*i*_) in the cardiomyocytes of mice. The results illustrated that [Ca^2+^]_*i*_ was upregulated as a result of the miR-377 overexpression, while the simultaneous overexpression of Rcan2 negated the upregulation of [Ca^2+^]. Meanwhile miR-377 inhibition led to reduced [Ca^2+^], and simultaneous treatment of miR-377 inhibitor and si-Rcan2 obviously promoted [Ca^2+^]_*i*_ versus miR-377 silencing alone (Figure 5(a)).

Moreover, miR-377 mimic alone elevated levels of miR-377 and CaN and diminished the Rcan2 expression, while the co-overexpression of miR-377 and Rcan2 reversed the effects of miR-377 mimic alone. miR-377 inhibitor alone diminished levels of miR-377 and CaN and enhanced the Rcan2 expression, while simultaneous depletion of miR-377 and Rcan2 negated the effects of miR-377 inhibitor alone (Figures 5(b) and 5(c)).

Further, miR-377 mimic alone impeded cardiomyocyte proliferation and augmented cardiomyocyte apoptosis, accompanied by elevated levels of inflammatory factors TNF-*α*, IL-6, and IL-8, whereas the simultaneous overexpression of miR-377 and Rcan2 still reversed the effects of miR-377 mimic alone. Consistently, miR-377 depletion alone enhanced cardiomyocyte proliferation and repressed cardiomyocyte apoptosis and inflammatory responses, the results of which were reversed by simultaneous depletion of miR-377 and Rcan2 (Figures 5(d)–5(f)).

Altogether, these results indicated that miR-377 inhibited Rcan2 and further restrict CaN via the Ca^2+^-CaN signaling pathway, thereby promoting cardiomyocyte hypertrophy in septic mice induced by CLP.

## 4. Discussion

Sepsis is regarded as a severe organ dysfunction caused by uncontrolled host responses to infection, presenting with major symptoms such as myocardial injury [20]. Under the condition of pathological hemodynamic overload, the dysregulation of specific miRNAs may change the cellular responses in cardiomyocytes and noncardiomyocytes, causing cardiac hypertrophy and heart failure [21]. Notably, miR-377 is known to possess the ability to target multiple genes and exhibit involvement in cardiomyocyte apoptosis [14]; yet, only a handful of studies have investigated its effect on sepsis-induced cardiomyocyte hypertrophy. Thereafter, we investigated in the present study the impact of miR-377 on myocardial hypertrophy caused by CLP-induced sepsis and elucidated the downstream mechanism.

An ever-increasing number of studies have indicated that miRNAs play critical roles in cardiac development and disease [22, 23]. Moreover, alterations in the miRNA expression in the hearts of hypertrophic mice are unraveled to be similar to those in the idiopathic end-stage failing human hearts, which suggests that miRNAs may exhibit molecular signatures of cardiac hypertrophy and have a role to confer in the pathological process of cardiac disease [24]. In addition, miRNA profiling studies have demonstrated that the level of specific miRNAs undergo progressive changes during the progression of cardiac hypertrophy caused by pressure overload [25]. Consistently, Care et al. illustrated in their study that inhibition of miR-133 in vivo cause sustained cardiac hypertrophy, indicating it is a key regulator of cardiac hypertrophy [26]; meanwhile, Huang et al. have revealed the correlation between the miR-221 expression and myocardial hypertrophy and fibrosis [27]. The topic of our focus, miR-377, was similarly associated with changes in cardiomyocyte apoptosis [14], while its effect on cardiomyocyte hypertrophy caused by sepsis remains to be explored. Expanding on current information, findings obtained in our study demonstrated that miR-377 could promote cardiomyocyte hypertrophy in CLP-induced sepsis in vivo, which highlights its negative-regulatory effect on cardiac hypertrophy.

Furthermore, bioinformatic analyses and mechanistic experimentation in our study revealed that Rcan2 is a downstream gene of miR-377, which was poorly expressed in the cardiac hypertrophy tissues of septic mice. Originally, Rcan2 was originally recognized as a thyroid hormone-responsive gene in human skin fibroblasts [28] and then highlighted as a mediator of CaN [29, 30]. More interestingly, alteration in Rcan2 levels was previously found to be correlated with the development of tumor [31], whereas Rcan2 is also known to regulate the obesity progression via a mechanism-independent of leptin signaling [32, 33]. Interestingly, the RCAN protein family has vital roles to confer in regulating inflammation [34]; for example, Rcan1 serves a negative regulator of inflammation in response to respiratory tract infections [35]. Elaborating on the significance of Rcan2, our findings further illustrated that miR-377 could specifically bind to Rcan2 and negatively regulated the Rcan2 expression, and downregulation of Rcan2 could reduce the expression of inflammatory factors in myocardial tissues of septic mice via a CaN activity-dependent mechanism.

RCAN2 family could interact physically with CaN and further regulate Ca^2+^/CaN signaling pathway [16]. Besides, Ca^2+^/CaN signaling is triggered by Ca^2+^ entering the cell from the extracellular space, which play a regulatory role in multiple disorders [36]. For example, the pathogenesis of cardiac dysfunction is attributed by sentrin/SUMO-specific protease 1- (SENP1-) mediated mitochondrial abnormities, and the upregulation of SENP1 in diseased heart is regulated by Ca^2+^/CaN pathway [37]; Ca^2+^/CaN signaling is important for the treatment of neurological insults [38]. Our experiment results demonstrated that the impact of miR-377 on myocardial tissue is linked to the Ca^2+^/CaN signaling pathway, which implies that miR-377 may regulate other diseases. In addition, Wu et al. has demonstrated that miR-30s could regulate Ca^2+^/CaN signaling in cardiomyocytes [36], which indicates that other miRNAs may be also potential targets for the treatment of myocardial hypertrophy and need further investigate in the future research.

It should also been noted that apart from targeting Rcan2, miR-377 had previously been reported to modulate various genes such as VEGF, CD133, and SIRT1 [39–41]. Herein, whether other miR-377-mediated signaling pathways or mechanisms are involved in the promoting effects of miR-377 on cardiac hypertrophy still requires further investigations. On the other hand, the downstream effectors of the RCAN2-Ca^2+^/CaN signaling pathway in sepsis-induced cardiac hypertrophy remain to be established. An elevated expression level of Rcan2 has been highlighted to reduce CaN activity and thereby blocking the activation of the CaN-NFAT signaling in denervated gastrocnemius muscle [42]. Additionally in gastric cancer, the participation of Rcan2 in tumor progression has been associated with EGFR, nuclear *β*-catenin, MMP7, laminin-*γ*2, and VEGF [31]. These lines of evidence indicate the necessity of future exploration of possible downstream mechanisms of the RCAN2-Ca^2+^/CaN signaling pathway. Moreover, miRNA profiling could be done in future studies to explore more potential miRNA with key roles in sepsis-induced cardiac hypertrophy.

## 5. Conclusions

To sum up, we found that miR-377 was a significant mediator of Ca^2+^/CaN signaling pathway and could regulate the activity of CaN by targeting Rcan2. Thus, our study provides interesting targets and biomarkers for novel strategies of the management of cardiac hypertrophy induced by sepsis.

## Data Availability

The datasets generated/analyzed during the current study are available.

## Abbreviations

miRNAs: MicroRNAs
CLP: Cecal ligation-perforation
CaN: Calcineurin
Rcan2: Regulators of calcineurin 2
FBS: Fetal calf serum
LVPW: Left ventricle posterior wall
EF: Ejection fraction
HE: Hematoxylin and eosin.

## Supplementary Materials

Supplementary Materials Supplementary Table 1 Primer sequences for qRT-PCR.
