# Supplementary material for: The Overexpression of miR-377 Aggravates Sepsis-Induced Myocardial Hypertrophy by Binding to Rcan2 and Mediating CaN Activity
Source: Oxid Med Cell Longev. 2022 Oct 11;2022:6659183. doi: 10.1155/2022/6659183 (PMC9578796; doi:10.1155/2022/6659183)
Supplement: Supplementary Materials — Supplementary Table 1 Primer sequences for qRT-PCR. [file 6659183.f1.docx]

**Supplementary Table 1** Primer sequences for qRT-PCR

| Gene | Sequence |
| --- | --- |
| miR-377 | F: 5'-GAGCAGAGGTTGCCCTTG-3' |
|  | R: Universal primer |
| Rcan2 | F: 5'-CAAGAGCGATCTCACCCCTC-3' |
|  | R: 5'-ATGAAGTAGGCGTCTCCCCT-3' |
| CaN | F: 5'-ACTGGCATGCTCCCCAGCGGA-3' |
|  | R: 5'-GTGCCGTTAGTCTCTGAGGCG-3' |
| U6 | F: 5'-CTCGCTTCGGCAGCACATATACT-3' |
|  | R: Universal primer |
| β-actin | F: 5'-ATTGCTGACAGGATGCAGA-3' |
|  | R: 5'-GAGTACTTGCGCTCAGGAGGA-3' |

Note: F, forward; R, reverse; miR-377, microRNA-377.
